# Attentional bias modification in reducing test anxiety vulnerability: a randomized controlled trial

**DOI:** 10.1186/s12888-017-1517-6

**Published:** 2018-01-05

**Authors:** Wenpeng Cai, Yu Pan, Huangyangzi Chai, Yi Cui, Jin Yan, Wei Dong, Guanghui Deng

**Affiliations:** 1 Faculty of Psychology and Mental Health, Second Military Medical University, Xiangyin Road 800, Shanghai, 200433 China; 2 Department of Medical Psychology, General Hospital of PLA, Beijing, 100853 China; 3 Department of Engineering and Information, Nanjing City Vocational College, Nanjing, 210038 China

**Keywords:** Test anxiety, Anxiety vulnerability, Attentional bias modification, Salivary amylase, Behavioral training, Dot probe, eStroop

## Abstract

**Background:**

A tendency to selectively process a threat to positive information may be involved in the etiology of anxiety disorders. The aim of this study is to examine whether attentional bias modification (ABM) can be used to modify high test-anxiety individuals’ attention to emotional information and whether this change is related to anxiety vulnerability.

**Methods:**

Seventy-seven undergraduates were included: 28 individuals received a 5-day modified dot probe task as ABM training, 29 individuals received a 5-day classic dot probe task as placebo, and 20 individuals did not receive an intervention between the two test sections. In addition to the measure of biased attention, salivary α-amylase (sAA) and the visual analogue scale of anxiety were assessed as emotional reactivity to stress.

**Results:**

A repeated measurement of variance analysis and paired sample *t*-test indicated that the ABM group showed a significant change in attentional bias scores after the 5-day training, whereas there were no changes in the attentional bias scores in the placebo or waiting list groups. Importantly, anxiety vulnerability with attention to threats was significantly decreased in the training group.

**Conclusions:**

These results suggest that attentional bias toward threat stimuli may play an important role in anxiety vulnerability. The attentional bias modification away from the threat is effective for the individuals preparing for an exam.

**Trial registration:**

This trial was retrospectively registered on June 22, 2017 with the registration number ChiCTR-IOR-17011745 and the title ‘Attentional Bias in high anxiety individuals and its modification’.

## Background

In general, anxious individuals preferentially allocate their attention towards threatening information in the environment over non-threatening stimuli, a pattern that is not detected in non-anxious individuals [1]. A range of evidence has suggested that this attentional bias may play an important role in the development and maintenance of anxiety and fear [2, 3].

Studies in non-anxious populations indicate that systematic training to attend to threats can increase susceptibility to stress [4, 5]. Moreover, several researchers have managed to reduce the attentional bias in anxious individuals using computerized attention tasks in which participants are trained to avoid the threat [6–8]. A modified dot-probe task was used in these studies. Two stimuli, including one threatening stimulus and one neutral stimulus, are typically involved and simultaneously presented. Following the removal of the stimuli, a target probe appears in the location previously occupied by one of the stimuli. Participants are instructed to determine the orientation of the dots by pressing one of two pre-specified buttons as soon as possible. The probes are more likely to appear in the location of the non-threatening stimulus, referred to as an attentional bias modification (ABM), which has been demonstrated to reduce anxiety and stress responsiveness at a medium effect size (d = 0.61) in a meta-analysis by Hakamata et al. [9]. However, more recent meta-analyses have failed to yield consistent findings regarding the beneficial effects of ABM on symptoms [10–13], and the effect sizes may be smaller than previously reported [10].

In contrast to a specific psychiatric disorder, test anxiety (TA) refers to “the set of phenomenological, physiological, and behavioral responses that accompany concern about the possible negative consequences or failure on an exam or similar evaluative situation” [14]. According to Zeidner, test anxiety is a combination of three facets: cognitive, physiological, and behavioral [15]. Cognitive symptoms include self-deprecating thoughts, expectations of failure, low self-esteem, and other off-task thoughts that detract attention from the task at hand. Physiological (i.e., emotional) symptoms may consist of an increased heart rate, perspiration, stomachaches, headaches, or other somatic symptoms that occur in response to evaluative situations. Finally, behavioral symptoms may include numerous observable behaviors, such as looking around the room, fidgeting, or chewing fingernails and pencils [16]. Many studies suggest TA contributes to impaired performances on examinations [17, 18]. This may be because individuals with TA are more likely to experience state anxiety across situations, which impedes focus and concentration in preparation for and during the examination. According to Lynn [19], individuals with high TA did not show longer response latencies to incongruent trials of words that denoted test anxiety or threat and were more likely to avoid threatening words rather than attend to them. However, additional research has suggested highly test anxious individuals exhibited an attentional bias to threat information [20, 21]. In this case, the effect of ABM on test anxiety individuals appears to be an interesting and valuable topic. Regrettably, few studies have addressed this issue. First, Dandeneau and Baldwin aimed to train a positive cognitive habit that would buffer against social and performance threats, thus making students less vulnerable and more resilient to rejection. Interestingly, participants in the ABM condition reported less interfering thoughts of being rejected when completing the anagrams task and an overall higher state self-esteem after having been rejected and experiencing failure [22]. McNally et al. subsequently reported reductions in self-report, behavioral, and physiological measures of speech anxiety using ABM [23]. Similarly, Hullu et at assessed the efficacy of a 10-week internet-delivered Cognitive Bias Modification with a focus on modifying both attentional and interpretive biases as a cost and time-efficient strategy to reduce social and test anxiety [24]. Moreover, self-reported social and test anxiety generally decreased from the pre-test to two-year follow-up.

Voogd’s research [25] is the first randomized controlled trial to assess the dot-probe and visual search training effects with up to one-year of follow-up. Three hundred forty adolescents were randomly assigned to dot-probe (DP) training, visual searching (VS) training, or placebo; the VS training was effective in reducing attentional bias in contrast to the DP training. As a result of the nature of online training, however, there were substantial drop-out rates and a lack of supervision. Therefore, a more optimal standard lab study is required to replicate the effect of ABM on test anxiety individuals. To explore the effect of ABM on test anxiety individuals, the current study recruited the participants preparing the College English Test 6 (CET-6). The CET-6 is a national English exam held twice per year in China. If college students fail to pass it, it results in substantial troubles for achieving their final degree and seeking jobs after graduation. In this case, majority of the candidates who are preparing for CET-6 must experience test anxiety.

Furthermore, there are several common limitations in previous ABM studies. First, the same behavioral paradigm was used in both the pre−/post-training and training tasks [26–28], which made it difficult to ensure whether the AB had been altered or it was only a result of a practice effect. Furthermore, several studies have indicated a relatively poor reliability of the dot-probe task [10, 13]. Many widely accepted paradigms have been used to assess attentional bias, such as the visual-probe task, emotion Stroop task, spatial-cueing task and visual-search task. Therefore, different AB paradigms were implemented in the pre−/post-training and training tasks in the following study. Second, several previous studies lacked a placebo training condition [27, 29]; thus, the direct effects of training towards threat could not be examined between different groups. Moreover, several studies have indicated that the control condition had a similar effect in reducing the AB or anxious symptoms as ABM [23, 30, 31]. Therefore, a no-training waiting list group was included in the current study to control for the placebo effects, positive expectations and demand effects.

Additionally, it has been widely accepted that salivary α-amylase (sAA), an oral cavity enzyme, is a powerful tool to indicate stress-reactive bodily changes, particularly the autonomic nervous system (ANS) [32]. This enzyme is rapidly increased in response to physiological and psychosocial stress [33–36]. The secretion of salivary amylase is directly stimulated by innervation followed by hormonal regulation in response to changes in serum noradrenalin levels. Therefore, the salivary gland acts more quickly and sensitively responds to psychological stress [37]. Compared with traditional stress makers, such as cortisol and catecholamines, salivary amylase has the advantages of noninvasiveness, a more significant increase, and a rapid response [35, 38]. Given the potential individual variability in responding to the same stressful condition, sAA were measured and integrated with the changes in attentional bias before and after the attentional bias modification.

Based on the previously reviewed literature, we aimed to determine whether attentional bias modification can be used to modify high test-anxiety individuals’ attention to emotional information and whether this change is related to emotional vulnerability, such as sAA reactivity. In addition to the placebo group, a waiting list group was employed to examine the direct effects of training.

## Methods

### Study design overview

The design included repeated-measures across two assessment times (pre-training and post-training). The pre-post assessment included physiological (salivary amylase), psychological (anxiety), and behavioral (attentional bias scores) indicators.

### Participant recruitment and allocation

**Fig. 1:**
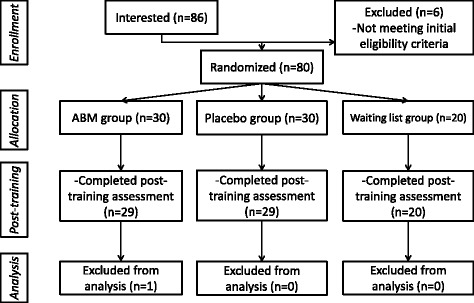
Flowchart depicting passage of participants

G*Power 3.1.9.2 (Heinrich-Heine-Universität, Düsseldorf, Germany) was used to determine the sample size. For an effect size of 0.4, an alpha probability of 0.05 and a beta probability of 0.8, the sample size required to detect a statistically significant difference is 20 participants per group. Given the loss of samples, ten additional individuals are required in the two intervention groups. Therefore, 80 participants were recruited from a medical university via advertisements placed in various settings (e.g., canteen, dormitory, and classroom building). During an initial phone and Internet instant message tool screen, the participants were told that the researchers were testing a new computer program designed to help individuals, who were preparing for the following CET-6, reduce test anxiety and develop healthy mental habits; moreover, the entire procedure, including the completion of the psychological scales and collection of saliva and computer behavioral data, would last one week. The Test Anxiety Scale (TAS) was used to assess test anxiety [39], and the Depression Anxiety Stress Scales (DASS) were used to assess the individuals’ depression, anxiety and stress states [40]. According to the CONSORT guidelines, the participants were randomly assigned to three groups (a: ABM group, b: placebo group and c: waiting list group) through the use of a computer-based random assignment program with a 3:3:2 allocation ratio. After the first day when they provided the baseline data, the participants were informed via e-mails regarding their assigned group (A, B or C) and the following programs. WC assigned the participants to an intervention based on a random allocation sequence in opaque, sealed, and stapled envelopes. The participants remained blind to the treatment hypotheses and the content of the other treatment groups. Moreover, the treatment allocation was concealed from the outcome assessor HC. As depicted in Fig. 1, three participants were excluded from the analysis for the following reasons: participant quit halfway through the study (*n* = 2), and there were too many incorrect responses during the eStroop task (*n* = 1). Thus, the final sample consisted of 77 undergraduates (57 males, 20 females), aged 18–25 years (M = 21.00, SD = 1.556). All participants were right-handed and reported normal color vision and normal or corrected-to-normal visual acuity.

### Procedure

**Fig. 2:**
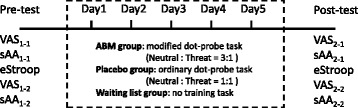
Repeated-measures study procedure

Figure 2 describes the sequence of events used in the procedure. After obtaining consent, the participants were instructed to rate their current anxiety using the visual analogue scale (VAS). A 10 cm line was divided into 10 equal partitions with the terminal labels “relaxed” and “anxious”. The participants circled the mark on the scale that most accurately reflected their current mood state. Scores ranged from 1 to 10, in which higher scores reflected a more anxious mood.

Salivary samples were subsequently collected using the hand-held monitor of sAA (Salivary Amylase Monitor, Nipro Co. Ltd., Japan), which was developed by analytical chemists Yamaguchi and colleagues. It had been reported to accurately and rapidly (within two min) assess individuals’ sAA levels associated with SAM activity [41]. The participants were instructed to rinse their mouths and wipe out all water. The collecting paper was then directly inserted into the oral cavity, and approximately 20–30 ml of saliva were collected from under the tongue over a period of 25 to 30 s. The reflectance 30 s after the initial time was automatically measured by this optical device. Thus, the measurement of the sAA level was completed in approximately two minutes. Salivary amylase measures were assessed four times in total (before and after the eStroop task in the pre- and post- training sections).

Besides, another central thesis in current study is that such attentional bias modification could serve to change individuals’ emotional vulnerability, resulting in a differential tendency to display elevated levels of anxiety mood state and sAA reactivity in response to a given stressor. To address this hypothesis, sAA reactivity and anxiety vulnerability were assessed for each eStroop task (i.e., pre-training and post-training). Scores were calculated by subtracting the pre-task state from the post-task state. For example, the pre-training sAA reactivity score for the first eStroop task was calculated by subtracting the salivary amylase before the first eStroop task (sAA_1–1_) from the salivary amylase after the first eStroop task (sAA_1–2_). Similarly, anxiety vulnerability prior to ABM was calculated by subtracting the anxiety visual analogue scales administered before the first eStroop task (VAS_1–1_) from the anxiety visual analogue scales administered after the first eStroop task (VAS_1–2_). The vulnerability scores after ABM used the salivary amylase and anxiety visual analogue scales from before and after the post-training eStroop task.

The participants completed behavioral tasks in a sound-attenuated psychology laboratory setting at a distance of approximately 60 cm from a 20-in LCD screen. Emotional Stroop tasks were used to assess the participants’ attentional bias in the first and last days, whereas dot probe tasks were used as attentional bias modification in the middle five training days. The ABM and placebo groups are instructed to perform the training between 12:00 p.m. and 9:30 p.m. every training day in the same laboratory.

### eStroop

One hundred twenty-eight emotional words, including 32 threat, 32 positive and 64 neutral words (Appendix A) were selected. The neutral words originated from the Chinese Affective Words System (CAWS), which was developed by the Psychology Institute of the Chinese Academy of Science [42]. In their study, 64 undergraduate students were instructed to rate the valence level, arousal level and dominance level for their word list using a 9-point Likert scale, which established the final CAWS. Similarly, the threat and positive words were exam-related and were rated by 30 undergraduates prior to the experience. In the current study, threat words (Valence_M ± SD_ = 3.04 ± 0.94), positive words (Valence_M ± SD_ = 7.80 ± 0.53), and neutral words (Valence_M ± SD_ = 5.49 ± 0.17) were presented in one of four colors (blue, yellow, red or green).

**Fig. 3:**
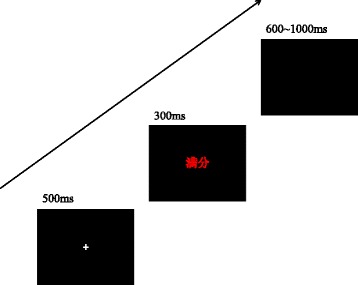
Sequence of events in the eStroop task

The current task was similar to that used by Taake et al. [43], which was divided into two different types of experimental blocks (threat/positive). In the Threat block, threat words and neutral words were randomly intermixed, with equal probability. The positive words and neutral words were in the Positive Block. Moreover, the emotional words were individually matched to neutral words for the frequency of use and length within each block. Two Threat and two Positive Blocks were run in the current task, and each block comprised 32 stimuli. The order of presentation was counterbalanced across participants. Each trial consisted of a white fixation cross against a black background for 500 ms, followed by the stimulus for 300 ms. The inter-stimuli interval randomly varied between 600 ms and 1000 ms (Fig. 3). The participants were instructed to maintain central fixation and discriminate the word color as quickly as possible by pressing the appropriate button (“S” for blue, “F” for yellow, “J” for red, and “L” for green), while ignoring the word meaning. Data were not collected until the participants performed more than 16 practice trials and the correct rate reached 75%. In current study, the eStroop task was used both as a measure of attentional bias and as a stress induction.

### Dot probe task

**Fig. 4:**
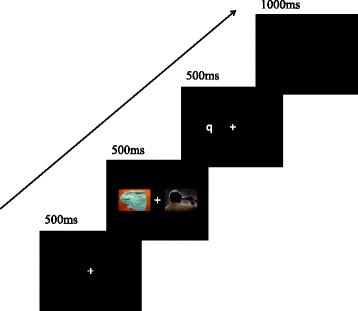
Sequence of events in the dot-probe task

Sixty threat and 60 neutral images were selected from the International Affective Picture System (IAPS) [44]. The neutral images included household objects, plants and neutral animals, whereas the threat images included natural catastrophes, dirty environments and aggressive animals. The dot-probe task consisted of 3 blocks, each of which included 120 trials. Following a 500 ms presentation of a fixation cross at the center of the screen, a pair of Threat-Neutral images was presented for 500 ms. The images were presented with equal distance to the right and left of the fixation cross (16.5 cm center-to-center). Following the removal of the images, a target display appeared for 500 ms, after which the screen went blank. The target display was the letter *p* or letter *q* and appeared at a distance of 8.5 cm to the left or right of the fixation at the location of the center of the left or right image (Fig. 4). The participants were instructed to determine the word by pressing one of two specified buttons. A new trial was initiated 1000 ms after target offset. The participants in the ABM group were presented with trials in which targets most likely replaced neutral images (75%). The participants in the placebo group were exposed to trials in which targets were equally likely to replace threat images. For both conditions, the neutral image was equally likely to appear on the left or right, and the target was equally likely to comprise the letter p or q. These variables were randomly mixed in presentation. Moreover, the participants in the waiting list group did not receive a task during these five days.

### Data analysis

The primary index of the training effect was derived by assessing the change in salivary amylase and visual analogue scales of anxiety over the 5-day training or placebo tasks. The changes in the response time of the eStroop were of specific interest. Only the reaction from correct hits was used in the following analysis. Moreover, RTs <300 ms or >1200 ms were excluded. We compared the pre- and post- training/placebo attentional bias scores (mean RT for Threat block – mean RT for Positive block). Positive bias values reflected attention towards threat, whereas threat values reflected the avoidance of threat.

The demographic variables were compared among the groups using analysis of variance (ANOVA) for continuous data and chi-square tests for categorical data. Attentional bias scores were subjected to 2 × 3 repeated measurement of variance analysis with time (pre-training, post-training) as a within subject factor and training condition (ABM, placebo, blank) as a between subject factor. In addition, changes in the salivary amylase and visual analogue scales of anxiety were assessed via univariate analysis. Pearson correlation was computed to explore the relationship between attentional bias toward threat and anxiety vulnerability.

## Results

### Clinical and demographic variables

The demographic characteristics of the participant groups are summarized in Table 1. There were no significant differences among the groups in the clinical or demographic variables, which indicates that randomization was successful.

### Change in attentional bias measured with eStroop task

**Fig. 5:**
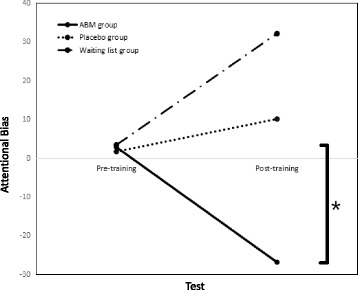
Attentional bias for the pre- and post- training

Table 2 presents the mean RTs of pre-training and post-training for the emotional (threat/positive/neutral) words in the threat and positive blocks for the ABM, placebo and waiting list groups. There was a nonsignificant difference between the groups on the attentional bias scores prior to the 5-day training (F(2, 74) = 0.006, *p* = 0.994, η^2^ < 0.01). A 2 (Test: pre-training, post-training) * 3 (Group: ABM, placebo, blank) repeated measurement of variance analysis was conducted on the attentional bias scores, with Group as a between-subject factor and Test as a within-subject factor. There were no test differences, F(1, 74) = 0.072, *p* = 0.789, η^2^ < 0.01. However, there was a group difference, F(2, 74) = 3.426, *p* = 0.038, η^2^ = 0.145. Moreover, the interaction of test and group was significant, F(2,74) = 3.323, *p* = 0.042, η^2^ = 0.082. Exploratory analyses were conducted via paired sample *t*-tests to examine group changes in attentional bias. As indicated in Fig. 5, the ABM group showed a significant change in the attentional bias scores after the 5-day training (2.73 ± 58.198 vs. -26.96 ± 46.549, respectively, t(27) = 2.077, *p* = 0.047, Cohen’ d = 0.392), whereas there were no changes in the attentional bias scores in the placebo group (1.59 ± 56.327 vs. 10.14 ± 71.737, respectively, t(28) = −0.493, *p* = 0.626, Cohen’ d = −0.09) or the waiting list group (3.30 ± 46.182 vs. 32.08 ± 53.001, respectively, t(19) = −1.862, *p* = 0.078, Cohen’ d = −0.416). It meant the attentional bias towards threat information could be altered by the attentional bias modification. The individuals after ABM training tended to have less attention on threats than before, but the other two groups remained unchanged.

### Effects of attentional bias modification on salivary amylase

Table 3 presents the mean sAA and sAA reactivity in the pre- and post- training sections. Changes in salivary amylase were initially tested as a function of the ABM procedure. Our dependent variables were the sAA values assessed directly after the training procedure (sAA_2–1_), and our covariates were the sAA values assessed at the beginning of the session (sAA_1–1_). After controlling for the pre-training sAA values, there were no group differences following the training (F(2,73) =1.947, *p* = 0.15, η^2^ = 0.051).

The sAA reactivity to stress was computed using sAA_1–2_-sAA_1–1_ and sAA_2–2_-sAA_2–1_. When controlling for the pre-training vulnerability, no significant vulnerability changes were identified (F(2,73) =0.567, *p* = 0.57, η^2^ = 0.015).

### Effects of attentional bias modification on anxious mood

Table 4 presents the mean VAS and anxiety vulnerability in the pre- and post- training sections. Similarly, changes in the visual analogue scale of anxiety were tested. Our dependent variables were the VAS values assessed directly after the training procedure (VAS_2–1_), and our covariates were the VAS values assessed at the beginning of the session (VAS_1–1_). After controlling for the pre-training VAS values, there were no group differences following the training (F(2,73) =1.193, *p* = 0.309, η^2^ = 0.032).

The anxiety vulnerability to stress was computed using VAS_1–2_-VAS_1–1_ and VAS_2–2_-VAS_2–1_. When controlling for the pre-training vulnerability, a trend group difference in the anxiety vulnerability was identified (F(2,73) = 2.765, *p* = 0.07, η^2^ = 0.07). To further examine this finding, pairwise comparisons were conducted, which suggested that individuals in the ABM group showed lower anxiety vulnerability to stress than individuals in the placebo group, *t* = −0.905, *p* = 0.026, Cohen’ d = −0.527.

We further assessed whether this anxiety vulnerability following training or placebo as a function was associated with changes in the attentional bias scores. A significant correlation between changes in anxiety vulnerability (post-training vulnerability minus pre-training vulnerability) and changes in the attentional bias score emerged only in the ABM group (*r* = 0.391, *p* = 0.040) and not in the placebo group (*r* = 0.194, *p* = 0.314) or the waiting list group (*r* = 0.122, *p* = 0.609).

## Discussion

Previous studies have indicated that attentional bias modification using a modified dot-probe task could influence cognitive styles and anxiety levels [2, 6, 8]; however, the effect of ABM on anxiety disorders has remained inconclusive, particularly with recent meta-analysis findings of dismissive appraisal as a viable clinical intervention [11, 13]. The current study aimed to determine whether ABM could alter the cognitive functions of attention processing in non-clinical (test anxiety) individuals and investigate the degree to which ABM reduces anxiety vulnerability.

The behavioral findings suggested that attentional bias toward threat information significantly decreased after the 5-day training away from the threat. The dot-probe task with a left-right orientation was used in the current study, which has been shown to be more effective than a top-down orientation [13]. In contrast to previous studies, the eStroop task used in the current study may indicate an automatic preferential allocation of attention resources to threat rather than task-related information. The attentional bias score can be assessed as the level of emotional stimulus content ability in interfering with the color naming, which causes a delayed reaction time compared with positive and threat words [45]. This modified Stroop paradigm was created to be task-wise unrelated to the training (dot-probe task), which was helpful to investigate how cognitive styles were influenced by attention training. Thus, the participants in the ABM group showed a significant reduction in attentional bias. They started to pay more attention to the positive words than the threat words after the 5-day training. In contrast, the participants in the placebo and waiting list groups tended to allocate their attention toward threatening stimuli with the approaching exam. These findings from the eStroop task may also be equally attributed to a bias in attentional engagement with or disengagement from the content of the threat stimuli [46]. After training, the participants in the ABM group showed an improved ability to shift attention from the meanings of negative words to task-related color naming, whereas an impaired disengagement account for attentional bias appeared in the placebo and waiting list groups.

Interestingly, the current findings linked attentional bias toward threat and anxiety vulnerability. The randomized block design effectively indicated that there were no differences among the three groups in anxiety, depression and stress prior to the training. After the 5-day attentional bias modification, the anxiety vulnerability with the attention to threats significantly decreased, which was not detected in the placebo and waiting list groups. These findings were consistent with previous studies [2, 4]. In both Eldar’s and Mathews’ studies, participants trained to attend to threat subsequently reported increased anxiety. These results suggested attentional bias toward threat information was implicated in the development of anxiety. The ABM could modify individuals’ attention processes and further influenced the reduction in anxiety vulnerability during a stress task.

Few researchers have directly explored the effects of attentional bias training on physiological markers of stress; however, preliminary results have been promising. According to the classical cognitive load theory, higher cognitive loads cause psychophysiological stress, as attention requires cognitive selection, and this effort elicits autonomic arousal [47]. Dandeneau et al. [48] reported basal cortisol was diminished after modified visual search training. Baert et al. [49] determined the indices of Heart Rate Variability (HRV) improved stress recovery by attention training via the dot probe. Moreover, Pilgrim et al. [50] showed attentional training elicits a paradoxical increase in cortisol and sAA reactivity. However, there were no significant changes in the salivary cortisol level after the 5-day training in the current study. One reason may be the lack of a well-validated psychosocial stressor. Emotional Stroop was not a novel task for the participants and failed to induce high cognitive loads and psychophysiological responses. Therefore, the sAA activity tended to decrease after the 5-day training in all three groups. Thus, a standardized task (e.g., Trier Social Stress Test [51]) is necessary in future studies.

Moreover, several recent studies have indicated a noncontingency-based training ABM task can be as anxiolytic as the typical contingency-based ABM training task [23, 52, 53]. Based on this finding, a waiting list group was included in the current study to control for potential placebo effects, positive expectations and demand effects. Consequently, there were no significant differences between the groups in anxious mood and physiological indicators, which is consistent with previous studies. Participants in the placebo group failed to improve their attentional bias, and the attentional bias scores of individuals in the waiting list group tended to increase (*p* = 0.07) at the post-training section. These findings suggested that the participants who received no training (or placebo training) started to develop negative cognitive processing with the approaching exam. These findings provided initial evidence that repeated concreteness training, not placebo, may have positive effects on attentional bias and naturally occurring symptoms in a test anxiety sample.

There are several limitations in the current study. First, limited participants were included in the waiting list group, which made it difficult to interpret the marginal significance. Practically, compared with the waiting list group, it is advised to set a group which trained individuals to attend to threat in the following studies. Thus, three types of dot-probe tasks should be included to investigate the effect of attentional bias modification on individuals’ attention processing. In addition, images from the International Affective Picture System rather than exam related images were used in the dot probe task. In most cases, it is exam-related information that induces the candidate’s negative feelings [54]. Therefore, it is suggested that standardized exam related images be used in future research. In this case, it may be more effective to modify high TA individual’s AB from negative exam-related information to positive exam-related one. Finally, there may be laboratory effects in this research. Previous studies have indicated larger effect sizes for anxiety symptoms at post-training in laboratory settings [13, 55]. Therefore, practical indices (e.g., test scores, sleep quality) appear equally critical. For example, exam performance has been shown to be improved by expressive writing [56, 57] and may improve high test anxiety individuals’ biology exam scores regarding writing about their thoughts about the upcoming exam. These issues indicate the need for systematic follow-up studies with a specific focus on practical indices on the eve of or exam day.

## Conclusions

In summary, the current study demonstrates that attentional bias modification away from a threat is effective for individuals preparing for an exam, and anxiety vulnerability can be modified by this simple cognitive intervention with the dot probe, which has been shown to alter attentional bias. Future studies in this area should investigate the effects of attentional bias modification on individuals’ performance on exam day, as well as the effects on cognitive processing over time.

## Abbreviations

ABM: Attentional Bias Modification
ANOVA: Analysis of Variance
CAWS: Chinese Affective Words System
CET-6: College English Test 6
DASS: Depression Anxiety Stress Scales
DP: Dot Probe
HRV: Heart Rate Variability
IAPS: International Affective Picture System
RT: Reaction Time
sAA: Salivary Amylase
TA: Test Anxiety
TAS: Test Anxiety Scale
VAS: Visual Analogue Scale
VS: Visual Searching
